# Barriers to clinical nurse participation in the internet-based home visiting program: a qualitative study

**DOI:** 10.1186/s12912-023-01651-9

**Published:** 2023-12-19

**Authors:** Jiahao Yu, Jianyuan Huang, Chunlei Li, Yongmei Zhuang

**Affiliations:** 1 Population Research Institute, Hohai University, Nanjing, 21100 China; 2 Zhongshan Hospital, Fudan University, Shanghai, 200032 China; 3 School of Nursing, Fudan University, Shanghai, 200032 China; 4 Jiangsu Health Development Research Center, Nanjing, 210036 China; 5 Jiangsu Provincial Medical Key Laboratory of Fertility Protection and Health Technology Assessment, Nanjing, 210036 China; 6 National Health Commission Contraceptives Adverse Reaction Surveillance Center, Nanjing, 210036 China

**Keywords:** Home care, Internet, Nurse attitudes, Barries, Qualitative study

## Abstract

**Background:**

Home visits are an important part of home care. With increasing demand and the rapid development of information technology, an increasing number of regions are experimenting with the use of information technology in home visits, hoping to meet the needs of more patients through technological interventions. However, most of the current studies have focused on patient health improvement through home visits, neglecting to consider the actual experience of nurses as service providers in participating in Internet-based programs. Thus, the purpose of this research is to explore what is holding nurses back from participating after the Internet has been added to traditional home visiting programs.

**Methods:**

This research was designed with an exploratory-descriptive qualitative analysis method. Semistructured interviews were used to collect information on barriers to nurses’ participation in the Internet-based home visiting program. Participants included 16 clinical nurses working in various hospitals in Nanjing, China. The thematic analysis method was used to analyze the information.

**Results:**

This research identified three themes and twelve subthemes that hinder clinical nurse engagement in the Internet-based home visiting program. The three themes included multiple barriers to individuals, different service modes, and emerging organizational problems.

**Conclusions:**

As a new form of traditional home visiting program in information society, Internet-based home visiting has many shortcomings in the overall program design and service management specifications. For more patients living at home to receive quality care services, it is necessary to take more effective measures to encourage nurses’ participation at three levels: nurse demand, service process, and organizational management.

**Supplementary Information:**

The online version contains supplementary material available at 10.1186/s12912-023-01651-9.

## Introduction

The aging of the population and the increase in the number of people with chronic diseases and disabilities requiring home rehabilitation have become global phenomena [1]. On the one hand, increasing aging has led to a surge in demand for in-home care services for many elderly people surviving illnesses [2]. On the other hand, the efficiency of public health service delivery in modern healthcare systems is low [3]. Hospitals discourage patients from staying in the hospital for too long to increase bed turnover and reduce the burden on medical insurance, leading to a rapidly growing demand for rehabilitation care services for a large number of postoperative patients [4]. In this context, home visiting programs, which are an important component of home care, are considered a solution for discharged patients and special populations who suffer from illnesses and have limited mobility to obtain continuous health management and support outside health care facilities.

The home visiting program is a long-term care strategy for providing specialized health or care services in the home environment [5]. Services in traditional programs consist of monitoring patients with chronic conditions, maternal or newborn visits, and extended care for patients discharged from the hospital. These orders are usually referred by social security departments or community medical institutions, and nurses visit patients in their homes at certain time intervals [6]. In past practice, home visiting programs have led to many positive health outcomes, whether to help individuals manage acute or chronic illnesses [7, 8] or to enhance their overall health [9]. In particular, it has been shown to be effective in reducing hospitalization and mortality rates in the populations served [10] and in meeting a large number of previously unmet medical needs [11]. With the enhancement of digital technology, telemedicine and online health information have provided patients with greater access to healthcare services [12]. Social security agencies in many countries are beginning to adopt digital means of service response, and a large number of Internet-based devices are being used by people and families. Unlike the regular visits of previous home visits, technology intervention allows patients to have a better service experience. Their needs are responded to more quickly, and problems are addressed in a timely manner. However, few people have noticed that nurses participating in the program are receiving an increasing number of home visit requests referred by electronic devices such as nursing platforms and panic buttons [13]. This not only poses a challenge to the nurses’ work but also places new demands on the continued operation of the entire home visiting program.

The home visiting program in China started relatively late, but its development process was consistent with that in most countries in the world [14]. In the late 1990s, home visiting services in China were mainly provided by community health care nurses. Limited by medical conditions and resources, services at that time mainly involved simple disease prevention, rehabilitation, health education, and family planning guidance [15]. In addition, Chinese health care workers preferred to take up employment in general hospitals, which led to a significant shortage of practitioners in community health care facilities. Most community nurses are more likely to wait for patients to arrive at the institution or use telephone visits [14]. Meanwhile, home visiting services in China used to include extended care services with hospitals. However, these services were only available for institutionalized discharged patients and were strictly limited to the area where the hospital was located, making the actual volume of services very sparse. Such health care programs are clearly unable to cope with the demand for home services arising from the rapid aging of China [16]. In response, the Chinese government launched an Internet-based home visiting program in 2019 in selected regions and rolled it out nationwide in 2020 [17].

The Chinese government defines this program as a “online application, offline service” model based on the traditional home visit program. Relying on information technology such as the Internet, in which the elderly, the disabled, patients with chronic diseases and patients in rehabilitation can invite clinical nurses working in professional medical institutions to visit. [18]. In view of the special nature of home visits, the Government has stipulated that the services that can be covered by the program must meet three conditions: (1) high demand for services; (2) easy to operate and implement; and (3) low medical risk. Nurses participating in the program are recruited by their institutions, and specialized government certifications (the multi-Practice Certification and internet healthcare qualification) are required after enrollment. They could choose to participate or not according to their own wishes. Nurses who participate in the program and actually provide services will be paid according to the content of their care. In this program, the nurse actually becomes the decision maker and implementer of the patient’s care plan. While providing professional nursing care to patients, the overall quality and self-worth of the nurses is further enhanced. In the specific operation process, after the patient asks for visiting through the application, smart platform, or emergency call, the technical operator dispatches the order to an institution that is relatively close to the patient. The agency sends it to the nurse based on the order demand, and the nurse chooses whether to respond or not based on his or her time and work schedule. This service is considered to be an effective adjunct to traditional programs [18]. But this service does not have access to any health insurance plan for the time being.

However, for the government and patients, this service allows for a wider use of health care resources to meet home care needs and can also increase the income of nurses and improve the social recognition of the nursing profession. However, as a major participant in the program, nurses’ work in the home care environment is extensive and complex, and Internet-based home visits further increase the risk of nurses’ work [19]. As a service model for traditional home visits in a new technological environment, Internet-based visits naturally inherit some of the work risks in the traditional model. First, the participating nurses are exposed to the risk of differences in the work environment. Unlike services delivered in an institution, home care is a more complex environment [20]. Nurses must find ways to work under unpredictable working conditions, such as the lack of a strictly sterile environment and assistive devices and sudden changes in patients’ physical indications. Second, nurses also need to bear traffic risks during their attendance [21]. Additionally, the additional workload associated with home visits makes it difficult for nurses to obtain adequate rest or update their knowledge [22], and this fatigue has led many nurses to report job burnout. In addition to these aforementioned risks, Internet-based home visits pose some new problems. For example, in traditional home visits, nurses mostly provide long-term services to patients they know well. Not only does the nurse have a better understanding of the patient’s health status, but both parties also have a stable nurse‒patient relationship [23]. In contrast, in Internet-based home visits, nurses not only receive more demand for their services but also may continuously meet new patients they have never seen before. This poses a significant challenge to their work. Significant changes in the context of services result in only a few nurses eventually being willing to join this new program [20, 24]. Previous studies have reported some information about nurses’ willingness to participate in the program, but they mostly looked at factors such as nurses’ demographic factors, awareness of the program, and self-perception [25–27], and their conclusions are basically the same as the surveys conducted for traditional programs. A very small number of studies have used qualitative research methods to explore in depth the barriers to nurses’ participation in the program [28–30]. Unfortunately, they still followed the interviewing strategy of the traditional home visit format, ignoring the challenges posed to nurses by changing work patterns.

The adoption of Internet technology has clearly brought about sweeping changes in the work patterns of home visiting nurses. The purpose of this study was to explore, through an exploratory-descriptive qualitative study, the actual experiences of nurses in the new home visiting program and whether the addition of the Internet created new barriers for them. This study may provide a basis for improvement not only for ongoing Internet-based home visiting programs in China but also for other countries that are running such programs with caveats to consider. At the same time, we hope this study will draw more attention from researchers to nurses.

## Methods

### Program background

This research was conducted in Nanjing, Jiangsu Province, China. As one of the first regions in China to pilot the Internet-based home visiting program, Nanjing has established relatively complete norms and processes for specific services and has accumulated a great deal of experience with the services. Its specific norms and processes are as follows:

All institutions participating in the program must be brick-and-mortar medical institutions that have obtained a “Medical Institution Practice License” and already have family beds, visiting services, and Internet consultation services. Nurses participating in the program must have at least five years of clinical nursing experience or the technical title of medium grade. At the same time, nurses providing specialized nursing services must obtain a certificate of qualification for specialized nurse training in the relevant specialty. In addition, Nanjing also has clear requirements for the service recipients participating in the program. Only people with limited mobility, such as discharged patients, elderly people, patients in recovery, pregnant women, disabled people and terminally ill patients with limited mobility, can receive home care services through the Internet. In terms of specific service items, the region provides six main types of services, including common clinical care (intramuscular injections, specimen collection, etc.), specialty care (maternal and infant care, tracheotomy care, peripherally inserted central catheter (PICC) maintenance, etc.), rehabilitation care (respiratory function exercise instruction, etc.), Chinese medicine care, chronic disease case management and health promotion (stress injury prevention, stroke rehabilitation, etc.), and hospice care (narcotic drug use).

The specific process for a patient or resident to schedule a service includes six parts. (1) The patient logs into the system to select the service and submit it. (2) The platform assigns the order to a medical institution that can provide the service. (3) The institution and the patient were contacted to confirm the service item, condition and medical history. (4) The institution sends the order to the nurse who is qualified to provide the service. (5) The nurse visits the facility to provide the service. (6) After completing the service, the patient evaluates the nurse in the system, and the institution pays a return visit by phone to them within 24 h.

The specific process of the nurse visit includes 4 parts. (1) Verify the patient/resident’s identity and case information. (2) Conduct an assessment of the patient’s/resident’s condition. (3) Explain the risks to the patient or family and sign an informed consent form. (4) Complete the service.

### Research design

As the current understanding of clinical nurses’ participation in internet-based home visiting programs is far from adequate. An exploratory-descriptive Qualitative study design was used for this study [31]. Under the guidance of humanistic values, We try to restore the actual service situation through participants’ words, and summarize the factors that prevent them from participating in the program. The fourth author is a researcher at Jiangsu medical authorities. She contacted medical institutions in Nanjing participating in the Internet-based home visiting program and requested their assistance in selecting participants. All nurses who participated in the interview were informed of the purpose, methods, and uses of the research. Corresponding data were kept confidential, and sensitive information was removed.

### Sampling and recruitment

In this study, we recruited two types of clinical nurses (participating in the plan and not participating in the plan) by purposeful sampling to ensure the heterogeneity of the sample. The inclusion criteria for clinical nurses who are participating in Internet-based home visits are as follows: (1) Having qualification certification of home visit. (2) Participation in Internet-based home visits for at least 3 months. (3) Having good mental health and memory. For clinical nurses who are not currently participating in the program, the inclusion criteria are: (1) Having the qualifications to be a visiting nurse according to program guidelines, Whether or not they have applied to the government for qualification certification of home visit. (2) Currently not participating in the home visit program. (3) Having good mental health and memory.

At the beginning of the recruitment process, the Nursing Department or Internet Program authority collected nurses’ willingness to participate in the study and assisted in the selection of potential participants. The first author contacted the nurses by phone and informed them of their rights. To ensure the independence of the study, information about all nurses who participated in this research was not returned to their institutions. Participants were also informed that they could refuse to participate or terminate their participation at any time without any consequences. To ensure that enough information was collected, recruitment was a long-term process. When two participants didn’t emerge new themes, we stopped recruiting. Ultimately, a total of 16 nurses participated in the research.

### Data collection procedure

Based on building a systematic understanding of existing relevant research, we conducted an extensive discussion to form an initial interview outline. The outline was then presented to 2 nursing specialists for review. At the same time, two nurses were selected for preinterviewing. Based on the results of the experts and preinterview, the final outline was revised.

All interviews were conducted between October and December 2022. Semistructured interviews were used to collect opinions about nurses’ participation in Internet-based home visits. We agreed on specific interview times with participants in advance. To create a safe and quiet interview environment, we chose to conduct the interviews in a café away from their institutions and with a private room. Interviews were conducted primarily by the first and second authors, and no fourth person appeared at the interview site. Field notes and audio recordings were recorded during the interviews with participants’ consent.

The second author is a professor with much experience in interviewing, and he led the conduct of the interviews. Through his efforts, a good relationship with the interviewees was established. Meanwhile, during the interview, the researcher mainly listened to the interviewee’s point of view and observed their body language and facial expressions. The researchers did not interrupt the interviewees except when they needed to clarify their points or when the interviewees elaborated for a long time on points that were not related to the content of the research. At the end of the interview, both interviewers reflected on the current interview to improve the quality of the later interview. Each interview lasted between 31 and 56 min.

### Data analysis

Within 24 h of each interview, the fourth author independently transcribed the audio recordings. and sent the material to the interviewee for review to ensure that we had faithfully captured their experiences and perspectives.

Within 24 h of each interview, the third author independently transcribed the audio recordings. The first author checked his transcribed text against the field notes and sent the material to the interviewees for checking to ensure that we had faithfully recorded their experiences and perspectives. Qualitative data were managed by Microsoft Word software. All authors were divided into two groups to work together on coding and analysis. To minimize the coding process being influenced by subjective feelings during the interviews, each group consisted of one researcher who participated in the interviews and one who did not (Yu&Zhuang, Huang&Li). In the initial stage of coding, the research team developed a detailed coding framework based on their understanding of the research questions and findings from existing research after reading the interview materials several times. An inductive thematic method, which focuses on extracting, condensing, and summarizing words and themes that recurred in the materials, was adopted during the coding process. Regular discussions about extending the coding framework and code assignment were conducted. After the coding was completed, the two groups exchanged the coding results used to review the discrepancies and discuss the controversial code assignments among them. A final coding result was determined that was harmonized by all members.

### Rigor

This study adopts the general standards (credibility, confirmability, dependability and transferability) proposed by Guba and Lincoln to test rigor [32]. In order to enhance the credibility and confirmability, we established a good relationship with the participants and triangulated the qualitative materials in the process of transcription, analysis and induction. To ensure the dependability of the study, we carefully designed the study and invited two experts outside the team to review it. To establish the transferability, we strictly followed the standardized reporting guidelines (COREQ checklist), and recorded the research process, sampling methods and the basic information of the final participants in detail.

### Ethics

This research was conducted in strict compliance with the ethical guidelines of the Declaration of Helsinki. Additionally, the corresponding procedures of the study were approved by the Academic Review Board of the Population Research Institute of Hohai University (No. 1098–3085), and the ethical legality of the survey process was regulated by this regulation. All participants volunteered for the interview and signed a written informed consent form.

## Results

### Participant information

A total of 16 nurses were interviewed in this research, and Table 1 provides the basic attributes of the participants. All participants were women with a mean age of 33.8 years (range from 23 to 51) and an average of 12.8 years of care experience. More than half of the participants held a professional title at the primary level or higher (56.2%) and had a specialist nursing qualification (62.5%). Eleven participants were still participating in the Internet-based home visiting program, with an average participation time of 12.5 months. Two participants were enrolled in the plan but are no longer serving, and 3 participants were never enrolled in the plan.

**Table 1:** Demographics of participants

Characteristics	n = 16 (100%)
Age (years), mean (SD)	33.8 (8.0)
Gender, n(%)	
Female	16 (100)
Education status, n(%)	
Associate degree	5 (31.2)
Bachelor	6 (37.5)
Higher education and above	5 (31.2)
Professional title, n(%)	
Primary title	4 (25.0)
Medium-grade professional title	7 (43.8)
Senior professional title	5 (31.2)
Specialist nursing qualification, n(%)	
Yes	10 (62.5)
No	6 (37.5)
Nursing experience (years), mean (SD)	12.8 (7.5)
Groups, n (%)	
Participation in Internet-based home visitation programs	11 (68.8)
Participation time (months), mean (SD)	12.5 (3.1)
Previous participation in Internet-based home visitation programs	2 (12.5)
Participation time (months), mean (SD)	8.5 (3.5)
Did not participate in the Internet-based home visiting program	3 (18.3)

### Themes

Among the barriers to clinical nurses’ participation in Internet-based home visits, the following 3 themes were identified: (1) Multiple barriers to individuals; (2) Completely different service modes; and (3) Emerging Organizational Problems. Meanwhile, 12 subthemes were identified in this research, as shown in Table 2.

The three themes were all about barriers encountered by clinical nurses in the process of participating in the program or in providing nursing services, but the themes had different emphases. The first theme focuses on the subjective willingness of nurses to participate in the program or not for personal considerations; the second theme focuses on the practical difficulties encountered by nurses in the process of service delivery; and the third theme focuses on the operational obstacles at the organizational level due to inadequate systems and standards.

**Table 2:** Themes and subthemes

Themes	Subthemes
Multiple barriers to individuals	1. More serious security risks; 2. Difficult to achieve a work-life balance; 3. Professional values not be respected; 4. Prone to psychological risks.
Different service model	5. Unfamiliar patients; 6. Non-standardized environment; 7. Unpredictable medical risks; 8. Lack of trust in communication.
Emerging Organizational Problems	9. Standards and norms not established; 10. Lack of team support; 11. Forced participation; 12. Imperfect platform.

#### Multiple barriers to individuals

Internet-based home visits provide a wider and more convenient way for the public to obtain nursing service. Compared with the traditional visits, the new program greatly increases the workload of nurses and makes them face more personal challenges. These challenges have had an impact on the motivation of nurses to participate in this program. Nurses will first consider whether participating in the plan will harm their personal safety and whether it meet their needs and expectations. For those nurses already in the program, they also encountered many barriers to providing care.This topic includes the following subthemes: more serious security risks, difficult work-life balance, professional values not respected, and prone to mental disorders.

##### Subtheme 1: more serious security risks

Unlike providing services in an institution or only to patients in the community, Internet-based home visits require nurses to travel to a relatively remote and unfamiliar area to provide services. This makes them vulnerable to road conditions, weather, and other reasons, as well as concerns about the safety of the commute and fatigue from the long distances involved.

N3: “Because many elderly live in the old quarter, the traffic there is not very good and many of the roads are very narrow. I’m not good at driving, so I worry about traffic accidents every time I go.”

N8: “Once I went to a community in the west of the city, it took me thirty minutes to ride my electric bike, and just met the heavy rain, I almost slipped on the road. When I arrived at the entrance of the district, the security guard would not let me in, and the family did not answer the phone, so it took about half an hour to get in. Such a round trip makes me physically and mentally exhausting.”

In addition, most of the nurses participating in the program are women. Although there are two nurses for each visit, the provision of services in a private environment worries them about their personal safety. They believe that if the government wants to carry out Internet-based home visits, the personal safety of nurses should be guaranteed first before more people are encouraged to join.

N2: “I sometimes wonder how I can protect myself when they close the door. However, there is no good way to do it.”

N6: “Sometimes I get scared when I receive an order because I do not know what kind of situation I will fall into. After all, there are always times when everyone is in a bad temper and gets angry.”

##### Subtheme 2: difficult to achieve work-life balance

The government requires nurses participating in the program to have more than five years of nursing experience, but these nurses have often become the main force of hospitals. This means that they are required to provide home visiting services in addition to the heavy nursing workload of the hospital and the burden of caring for their families. They were concerned that their limited time and energy would not be enough to accommodate these roles at the same time. Many participants said that online nursing takes up their rest time, which had a significant impact on their willingness to participate in the program in the long term.

N9: “Home visits can provide a lot of convenience for patients, and we are willing to do it. However, we usually have a large workload, and it is common to work overtime. When receiving a home visit order, i must consider our schedule and the distance to the destination.”

N12: “In the past, when I was involved in home visits, the times were fixed. For example, I would visit every other week after patients were discharged from the hospital. I could arrange my schedule in advance. However, now it is not possible at all. you do not know when the orders are coming.”

Some participants also mentioned that home visits took away from their participation in family life, which caused dissatisfaction among their families.

N10: “Now I cannot go home to my children after work because I need to provide home care for my patients. This makes my family have a lot of complaints about me.”

N15: “They want me to spend more time at home instead of going out to do these ‘public welfare activities’.”

##### Subtheme 3: professional values not respected

Under the current system, Internet-based home visits in China are charged at a fixed rate. This is regardless of the distance traveled or the qualifications of the nurse providing the service. Some participants felt that such fees did not motivate senior nurses to participate in the program and ignored the value of nursing services.

N4: “Nurses with different qualifications should charge different nursing expenses. For example, nurses with senior professional titles charge 200 yuan per service, and primary nurses charge 150 yuan per service. This will increase everyone’s motivation.”

N9: “Patients can choose nurses with different qualifications for services on the platform, and the service fees should be different for nurses with different qualifications, which better reflects the professional value of nurses.”

In addition, nurses generally felt that the brokerage fees charged by the platform were so high that they were confused as to whether the program was a nursing service or a platform service. This goes against the promise of increased compensation that was advertised to attract nurses. Many participants refuse to continue providing services.

N5: “The platform settles for a very low wage and it is getting lower…. I used to get paid 200 yuan for 3 hours of home visits, now I only get 300 yuan a day at most.”

N11: “We are now confused whether this is a government program or a corporate program. The fees charged by the platform are too high. If a project patient pays 140 yuan for service, we can only get 60%, and we basically do not make any money after travel expenses.”

##### Subtheme 4: prone to psychological risks

This is different from institutional care and traditional home visits, where nurses have discretionary authority. Nurses act on patients’ orders and can only provide advice on further treatment for patients when providing internet-based home visits. Some nurses reported that they empathized with the pain suffered by their patients. If patients’ families do not take effective measures and lead to deterioration of patients’ health, they can easily self-attribute and develop psychological problems.

N1: “Most of the patients who need home visits are in poor health, and some of them are in great need of treatment in specialized institutions. However, we do not have the authority to ask families to do so…. After a long time of contact, it is easy to feel their feelings, but there is nothing we can do about it.”

N7: “One time when I went to provide sputum suction service, the patient was already in bad shape. I suggested that the family call hospice services, but they did not take my advice…… Two days later, the director told me this patient had passed away, and I was very sad that he could have left in a more comfortable way.”

#### Different service models

In addition to the direct obstacles to nurses, the service mode of internet-based home visits has also changed greatly, especially in the service object and content. Nurses believe that these changes have given birth to new service risks and hindered their willingness to participate in it for a long time. This theme includes the following subtheme: unknown patients, humble working environment, unpredictable medical risks, and lack of trust in communication.

##### Subtheme 5: unfamiliar patients

Unlike traditional home visits that face discharged patients or patients in the community, Internet-based home care is almost unfamiliar to nurses. Most of the participants reported a certain amount of stress in service for patients they had not encountered before. This stress comes from the concern of hiding the patient’s condition on the one hand and is related to the inability to carry out care based on the patient’s condition on the other hand.

N3: “I’m concerned about the possibility of a patient hiding the history of infectious diseases. If they conceal it, even if we take standard protective measures, there will still be a risk of occupational exposure.”

N2: “On one occasion, I went to the patient’s home and found that the patient was in an abnormal mental state. He was agitated and uncooperative. I did not know this from the patient’s records and did not be informed it when I talked to the family on the phone.”

N10: “There are times when patients are in bad shape and their families are anxious. We have no way to obtain the patient’s medical history on the spot. The information on the platform is also very limited, which brings great troubles to our work.”

##### Subtheme 6: humble working environment

Providing services in the home means that nurses move away from the standardized environment of a hospital and into the relatively humble environment of care. Nurses need to expend more effort than usual to complete a service, which makes home visits difficult. At the same time, traditional home visits are almost noninvasive and do not generate medical waste that easily causes injuries, such as sharp instruments. In the new program, the waste needs to be brought back to the institution. It is easy to contaminate the nurse on the way back to the institution. This kind of humble working environment deeply worries participants.

N7: “The bed at home is not like a hospital with an infusion stand. Last time I did bladder irrigation for a patient, it was a family member holding it up for me on the side, which was not very convenient.”

N9: “You need light to do the operation so that the view is clear. Some homes place patients in shaded rooms, and the lights are not bright enough, which greatly affects the operation.”

N11: “According to stipulations, we are required to bring back used sharps and other medical waste to the hospital after the care. However, we were not provided with a portable sharps box, and needle stick injuries could occur on the way back. This is a safety risk.”

##### Subtheme 7: unpredictable medical risks

The medical risks involved in the home care process are also an important factor that prevents nurses from participating in the program. From the service components mentioned above, approximately half of the services are invasive operations. The home environment does not have strict aseptic conditions. This means that nurses must give more consideration to the safety of the nursing process. However, when providing care to unfamiliar patients in a noninstitutional environment, the nurse cannot fully anticipate the risks that may arise, and they may not be related to the nurse’s personal competence.

N6: “After one of our hospital nurses changed the urinary catheter for a patient, the patient developed an infection and passed away…. His family members still come to the hospital to protest…. Now I tell myself not to do these services after seeing it.”

N7: “Intravenous injection is an invasive operation and needs to be performed strictly according to medical advice. How can we be sure of the authenticity of the prescription provided by patients? How can we guarantee the safety of drugs purchased by the patient’s family members themselves?”

N9: “Injections may seem simple to patients and families, but there are risks involved. If a serious infusion reaction or allergy develops, it is difficult for the nurse to prove that she is not responsible.”

##### Subtheme 8: lack of trust in communication

As a professional service, knowledge between the nurse and the patient is not reciprocal. Therefore, the interaction between the two is particularly important. However, not only is the patient unfamiliar to the nurse, but the nurse is also unfamiliar to the patient. Patients may be concerned about the quality of care and may not cooperate with the nursing. Such an attitude not only makes the nurse feel stressed but also does not contribute to a good relationship between nurses and patients.

N5: “During a home visit, the catheter I had placed in the patient had a leak. The family members thought that our operation was incorrect and kept asking for a senior nurse to be sent over to reoperate. This made me feel bad.”

N4: “Rehabilitation is a long-term process, so compliance is very important for elderly patients with chronic illnesses. If they do not trust me, not only will it be a hindrance to my on-site nursing, but the end result will be poor if they do not strictly follow the required autonomy training after I leave.”

N16: “We cannot guarantee that patients will trust us completely. If there is miscommunication because of mistrust, who will bear the negative consequences in the end?”

#### Emerging organizational problems

Home visits are a special kind of nursing service, and nurses need effective support and understanding from their institutions and service platforms to improve the quality of nursing. Unlike traditional visits, which have developed a relatively well-established system of management and cooperation over a long-term practice, the new program not only lacks specific management norms, but also presents new organizational barriers to coordination and participation within the institution. The third theme records the organizational problems that nurses felt in the new program. This theme includes the following subthemes: regulations and systems not established, lack of team support, forced participation, and imperfect platform.

##### Subtheme 9: standards and norms not established

Currently, Internet-based home visits are still in their infancy, with no proprietary nursing standards and norms and no clear attribution of responsibility. In particular, how to deal with an emergency and help them smoothly adduce evidence is a major concern for participants.

N7: “How can we ask the hospital for help when we encounter problems during home visits? This requires the hospital to formulate a well-developed emergency treatment process and tell us how to deal with it, instead of simply following the previous home visit standards.”

N10: “The government and the hospital did not provide protection for us at all, which made me feel that I was operating illegally out of the hospital and that it must be all my responsibility after something happened.”

N14: “The fact that we go to a patient’s home for services means that we enter a situation where only two parties, me and them, are present. If there is a dispute, I am definitely at a disadvantage and no one will testify for me…… Cannot we be equipped with portable video recorders, just like the police?”

##### Subtheme 10: lack of team support

Several nurses pointed out a lack of support resources available to them in this program. In particular, nurses did not have a professional team to turn to, and they were left on their own to complete care services. This diminished their confidence in completing home visits.

N1: “In previous family visits, not only would nurses be involved, but family doctors in the community would also be there. Now that the family doctors are not paid for this program, they no longer show up. If I do meet a patient with a complicated condition, I use my personal social connections to ask for their cooperation. However, only if there is a family doctor there that I know.”

N4: “There are times when we need some medicines from the hospital that require a prescription from the doctor. However, they do not want to. The doctors think they are not part of the program and have no authority to do this. Even if I just need some glucose or anti-inflammatory medicine.”

At the same time, the provision of skilled nursing service in the home lacks the necessary assistive and emergency equipment. This poses a great challenge to the personal competence of the participants.

N9: “During the procedure of placing an indwelling gastric tube for our patient, I was unable to completely confirm that I had left the tube in my stomach after both procedures because of edema in his larynx. When I pulled out the stomach tube, I found some bloody liquid, which worried me about whether it hurt the patient’s lungs. I wish I had a visual laryngoscope.”

N15: “There’s also the question of how do I resuscitate a patient in serious condition if she falls into a coma or other emergency problem during nursing? We are not equipped with equipment such as an AED, let alone a resuscitation alert button that can be called for help at any time in the hospital.”

##### Subtheme 11: forced participation

In addition to the lack of standards and team support, participants also reported that they were forced to accept orders to improve hospital response rates for Internet services. This had never been seen in previous programs and greatly discouraged nurses from participating.

N12: “It is mandatory for our hospital to take orders, and it is linked to promotion. Only nurses who take more than five orders a year can be promoted.”

N8: “They have added this to the appraisal of nurses. If you are involved in the list of the program but do not provide service every month, your appraisal score will be lower and your bonus will be reduced.”

##### Subtheme 12: imperfect platform

Internet-based home visits are highly dependent on the information provided by the service platform. Regrettably, the current platforms do not perform their duties excellently. Nurses report that the platforms often have problems such as inaccurate positioning and lagging information push, which not only inconveniences nurses on visits but also increases the potential risk of disputes.

N1: “The platform offers navigation, but it is hard to get me to the right place…. not occasionally, but almost every time. This is very frightening in an emergency situation.”

N5: “The platform is very imperfect, not only for nurses but for patients as well. There are times when patients place orders but they do not show up in the nurses’ system.”

N13: “Right now this platform is a separate platform, it is not connected to either the national health system or the hospital’s medical information system. If it is a patient discharged from our hospital, I can take a picture of the patient’s previous medical history before I leave. However, if it is an unfamiliar patient, I have absolutely nothing to do.”

## Discussion

This research identified three themes in exploring the barriers to clinical nurse participation in Internet-based home visiting programs: multiple barriers to individuals, different service modes, and emerging organizational problems. As a whole, the lack of protection, understanding, and support for nurses is what ultimately leads to their worrying about participation in the new program.

At the individual level, nurses perceived that the Internet-based home visiting program exposed them to additional safety risks. They were concerned about encountering inclement weather or traffic accidents during their commute, which is basically the same as the findings of previous studies [19, 21]. At the same time, they are also concerned in this program that they might suffer threats to their personal safety by providing services to strangers in a private setting. The government should make it mandatory for institutions to provide nurses with necessary protective measures such as one-button locator alarm devices and work recorders to minimize the safety risks of the program and encourage nurses participation. In addition, nurses were concerned that their limited time and energy would not support them in adapting to multiple roles simultaneously. Previous studies have confirmed that excessive hours significantly reduce nurses’ willingness to engage in long-term nursing [30], and the involvement of new programs is making this issue more acute. To run this program for the long term, it is necessary to provide nurses with more flexible shift and break options. Institutions and platforms should consider factors such as the patient’s home distance, the nurse’s daily scheduling and the frequency of taking orders when dispatching orders. Avoid excessive order taking by nurses in order to increase their income, and ensure reasonable rest time for nurses. At the same time, reasonable compensation is not only a way to attract more nurses to the program but also a way to better reflect the value of nursing. However, past reports have indicated that many nurses are satisfied with the compensation they receive for participating in the program [22]. They simply compared the difference in the cost of care in institutions and hospitals, ignoring the additional expenses that nurses may incur [1]. In our research, nurses pointed out that not only did the platform not take into account the cost of promotion, the fees charged were almost equal to the wage nurses received. This behavior severely undermines nurses’ recognition of the professional value of nursing and reduces their motivation to participate in the program. The government should standardize the benefit distribution model of the program by means of the government’s own development of the platform or the promulgation of regulations on platform fees. This will not only further clarify the public health service attributes of the program, but will also be able to encourage more nurses to participate. We also found it difficult to ensure nurse autonomy in the complex and less regulated setting of home care [33]. The need for nurses to respect the autonomy and lifestyle of patients and their families and the inability to strictly require patients to do what is medically correct according to professional judgment [34] is seen as a moral pressure on nurses [35]. Under this pressure, nurses will be very prone to empathize with their patients. While a lack of empathy may reduce the effectiveness of treatment, excessive empathy may lead to nurses suffering from negative emotions such as frustration [36]. Therefore, on the one hand, the government and institutions can help HVNs make psychological preparations in advance through thematic lectures, case analysis and discussion, and scenario simulation exercises. On the other hand, they should provide HVNs with a long-term psychological counseling support program. Timely counseling work should be carried out after HVNs have encountered unexpected situations, so as to ensure that they do not suffer too much psychological distress.

At the service level, the Internet-based home visiting program further exacerbates the difficulty for nurses to provide services. The new program allows nurses to frequently meet unfamiliar patients and requires nurses to provide services to patients for the first time with a lack of information. These factors greatly increase nurses’ concerns about being able to complete quality care. Institutions should do their due diligence in taking a detailed past medical history of the patient and establishing an early warning system for home visit risk prevention to screen for possible risks. To provide HVNs with the information they need as much as possible, and remind them of possible risks in time. Clutter, sketchiness and mobility were the words most frequently mentioned by nurses in the work environment during Internet-based home visits. Past research confirms that the dynamic yet unpredictable environment faced by home care may cause nurses to feel rushed or distracted in the delivery of services [37]. With complex care programs and limited service time, the humble nursing environment hinders them from providing quality care to patients [38]. The absence of a strict sterile environment in home care also increases nurses’ concerns about home visits. A previous study found that contamination due to environmental factors in home care was the main cause of infection in patients [39]. Nurses may encounter a variety of safety hazards in patients’ homes, which hinders their strict adherence to infection prevention practices [40]. Apart from trying to avoid setting up items in the list of services that are complicated and difficult to operate, the Government also needs to design a home environment assessment checklist. HVNs should be allowed to conduct a systematic assessment of the patient’s health condition and their home physical environment after they reach the patient’s home, and the services that can be carried out should be decided on the basis of the assessment results. This is not only to reduce the pressure on nurses but also to maintain the safety of patients themselves. In addition, the interaction between the nurse and the patient is an important basis for the nurse to provide nursing service. The quality of the interaction will have a direct impact on the final quality of care and patient satisfaction with the nursing service [41]. In China, nurses have less professional trust than physicians. In particular, professional hierarchy is prevalent in patients’ minds. A patient may simply judge the rank a nurse may be in by her age. This mistrust of the nurse may be exacerbated when he perceives a mismatch between needs and rank. The lack of trust can greatly increase the difficulty of care by making patient compliance less likely. The government needs to use TV or newspaper news, the Internet and other forms to strengthen the publicity of the program. Institutions should increase training for HVNs’ professional skills and communication skills, and guide them to win the trust of patients through superb nursing skills and sincere communication. In addition, prioritizing the dispatch of nurses who are familiar with patients can also effectively reduce potential nurse-patient trust risks.

This research also sheds new light on the organizational barriers that Internet-based home visits can encounter, which are rare in traditional home visits. While information technology can bridge the time and space gaps of previous care delivery, it cannot be assumed that Internet-based home visits have not changed the nature of home visits, and the existing standards can be extended in this program. While the essence of nursing is the same, many factors, such as the way service calls are made and the environment in which nursing is provided, have changed. For example, lack of teamwork was the most cited barrier by participants. In contrast, in traditional home visits, advance preparation and enhanced learning can be relied upon to compensate for the lack of organizational support [42]. Not only are more challenging services required in the new program, there is far less team and equipment support than in the past. However, over time, nurses are relying less on specialists and trying to become all-rounders [34]. However, this conclusion is predicated on the premise that nurses need to experience all elements of practice required for patient needs [43]. This is almost impossible in new programs with so many service lines. Institutions where HVNs are employed should have a dedicated online support platform and experienced healthcare professionals on duty to assist HVNs with different conditions. At the same time, incomplete regulations and systems leave nurses with a lack of ways to protect themselves and seek help in emergencies. As a special category of services within the complex system of nursing, home services require more regulated and effective regulatory tools from the government and agencies [44, 45]. A case in South Korea has proven that the lack of policies and effective guidelines is one of the main reasons hindering nurses from participating in home visits [46]. It is important for medical authorities to introduce home visit management regulations that are in line with the nature of the Internet and to enhance training for nurses as soon as possible. Clarifying the rights and responsibilities of nurses through regulations is not only to make nurses less worried about the risks of care but also to better improve the quality of care. Some participants also pointed out the phenomenon of institutions forcing nurses to participate in the program by linking program participation to performance and promotion appraisals. Under the current premise of a severe shortage of primary care services, nurses’ human resources, and other healthcare infrastructure in China, participating in the program against nurses’ will to work would be detrimental to the fragile healthcare environment. It is important to respect nurses’ willingness and prohibit institutions from mandating nurses to participate in home visits. The number of nurses engaged in the service can be increased by encouraging willing retired nurses to participate in the program [47]. This research also identified barriers regarding the use of the platform, which have never been mentioned in previous studies. The purpose of introducing the Internet into traditional home visits was to reduce communication and health insurance costs and to improve the responsiveness and coverage of services [1]. However, the platform is still in the pilot phase, and the features have not been thoroughly developed. To better reflect the advantages of Internet-based home visits, developers should reduce the operational steps of the platform as much as possible and, on the basis of improving the basic functions of the platform (patient location and service dispatch) as soon as possible, connect the platform to the national integrated medical information network to reduce the learning costs of nurses and patients in using it and improve the efficiency of nursing services [48].

### Limitations

This research is a pilot study in China. Most of the clinical nurses who participated in the study were from general healthcare institutions, which implies a possible selection bias in the sample. However, this study downplayed contextual factors as much as possible in the interviews and writing, focusing on providing information on what new barriers nurses encounter in traditional home visits and Internet-based home visits. This may provide some ideas for other areas where the program is being developed.

## Conclusion

Internet-based home visiting programs are a new form of traditional home visiting, but many barriers are preventing nurses from participating in the programs in the early stages of implementation. The most significant and common issues include: multiple barriers to individuals, different service models, and emerging organizational problems. in order for the program to be implemented in the long term, effective measures must be taken to alleviate nurses’ concerns about the program and encourage their further participation. In order for the program to be implemented over the long term, effective measures must be taken to alleviate nurses’ concerns about the program to encourage further participation. Effective measures may include three areas. At the level of individual nurses’ needs, nurses should be provided with necessary security, such as one-touch alarms and portable recorders, adopting a flexible order-taking model and shift system, increasing nurses’ compensation for participating in the program, and providing nurses with long-term psychological counseling services. At the service process level, strengthen the review of patient information and medical history, avoid complex invasive nursing services in the home environment as much as possible, and help patients develop a proper understanding of the nursing model and role. At the organizational management level, introduce relevant policies and guidelines, establish effective internal teamwork mechanisms, prohibit institutional mandates on nurse participation, and continuously improve platform functionality.

### Electronic supplementary material

Below is the link to the electronic supplementary material.

Supplementary Material 1

## Data Availability

The datasets used and/or analyzed during this study are available from the corresponding author on reasonable request.
