# Supplementary material for: Barriers to clinical nurse participation in the internet-based home visiting program: a qualitative study
Source: BMC Nurs. 2023 Dec 19;22:486. doi: 10.1186/s12912-023-01651-9 (PMC10729343; doi:10.1186/s12912-023-01651-9)
Supplement: Supplementary file 1 — Supplementary Material 1 [file 12912_2023_1651_MOESM1_ESM.docx]

**Appendix 1 Interview Outline**

**Note：**

**The question numbered A is for the nurse who is participating the Internet -based home visiting program. The question numbered B is nurse who is not participating the Internet-based home visiting program.**

**Main problems：**

1. a.b. How do you understand the the Internet-based home visiting program?
2. a.b. Can you tell us the difference between it and the traditional home visiting program?
3. a. How do you feel after participating in the project? Does it meet your expectations?

b. Why don't you participate in the program？

1. a. Did you face any difficulties when you participated in this program?

b. What prevented you from participating in this program？

1. a. Does participating in this program have an impact on your daily work and life?

b. What impact do you think it would have on your daily work and life if you participated in this project?

1. a.b.What basic elements do you think this program should have?
2. a.b.Do you think there is anything that should be done to improve this program?
3. a. Will you continue to participate in this project?

b. Will you participate in the future?

**General problems：**

1. Can you explain this in detail?

2. Can you think of an example?

3. How do you feel about it?

4. What do you suggest?

5. Do you have anything else to add?
